# Reduced prediction updating shapes serial dependence in autistic traits

**DOI:** 10.1186/s12915-026-02518-6

**Published:** 2026-01-23

**Authors:** Antonella Pomè, Michael Wiesing, Eckart Zimmermann

**Affiliations:** 1 Institute for Experimental Psychology, Heinrich Heine University Düsseldorf, Universitätsstr. 1, Düsseldorf, 40225 Germany; 2 Event Lab, Department of Clinical Psychology and Psychobiology, University of Barcelona, Barcelona, Spain

**Keywords:** Sensorimotor serial dependencies, Prediction updating, Autistic traits, Virtual reality

## Abstract

**Background:**

Serial dependence, the influence of prior experience on current perception or decision, has typically been studied in static, perceptual contexts. Here, we investigate whether serial dependence reflects not just passive carryover but feedback-based updating of internal models, and how this process varies with autistic traits. In an immersive virtual reality penalty-kick task, participants kicked a ball that disappeared mid-flight and estimated its landing position. By laterally displacing the ball upon reappearance, we introduced trial-by-trial prediction errors.

**Results:**

We found that individuals with higher autistic traits showed larger prediction deviations, indicating mis-calibrated forward predictions. At the same time, their responses were more strongly shaped by those priors, and unlike lower autistic traits individuals, they did not down-weight reliance when distortions were maximal. This pattern suggests reduced flexibility in updating prediction use: priors were both less accurate and more rigidly applied. Classical stimulus and response history biases were unaffected by autistic traits, highlighting a specific impairment in prediction updating. Football experts, by contrast, combined low directional updating with near-zero prediction consistency, suggesting robust mappings that resist transient perturbations.

**Conclusions:**

These findings suggest that serial dependence in dynamic tasks reflects not only prediction formation but the flexible (or rigid) deployment of those predictions in the face of changing feedback. Our results highlight a distinctive rigidity in prediction weighting, rather than a general perceptual bias, in individuals with elevated autistic traits, and reveal contrasting stabilization strategies in domain experts.

## Background

Imagine trying to predict the path of a ball in mid-flight while running on a soccer field. In such dynamic situations, the brain does not passively respond to sensory input; it proactively anticipates the consequences of our own actions [1]. Predicting the sensory outcomes of self-generated movements is essential for interacting effectively with a rapidly changing world, especially under conditions of uncertainty. Rather than processing sensory information in isolation, the brain continuously generates internal predictions based on motor commands, prior experience, and contextual cues, and compares them to incoming sensory feedback (for a review see [2]). This predictive-feedback loop supports flexible motor control; perceptual continuity across movement (e.g., saccades, reaches); and ultimately allows for coherent action in noisy, ambiguous environments [3, 4].

A key mechanism enabling this loop is the efference copy, an internal signal representing planned motor output, used to forecast the sensory consequences of actions [5, 6]. These predictions help discount self-generated motion, stabilize perception during eye movements, and interpret ambiguous input considering expected outcomes [5–7]. Crucially, these internal models are not fixed: they are updated dynamically based on prediction errors, i.e., mismatches between expectation and sensory reality [8]. This updating process is vital for refining future predictions and maintaining sensorimotor adaptability [3, 8].

In autism spectrum disorder (ASD) and individuals with heightened autistic traits, multiple lines of evidence suggest that this predictive loop may function atypically. Predictive coding frameworks propose either a diminished reliance on prior information [9] or overly precise, inflexible internal models that resist updating in light of sensory feedback [10]. Both accounts imply a disruption in the brain’s ability to generate, maintain, and revise predictions, particularly when integrating self-generated actions with their sensory consequences.

Recent empirical work from our lab has pointed to efference copy dysfunction as a key mechanism underlying these predictive disruptions. In a spatial updating task involving two sequential saccades, we found that participants with higher autistic traits consistently mislocalized the second saccade target, indicating an inability to properly monitor the vector of their primary saccade by an efference copy signal [11]. Further experiments revealed that imprecise efference copy signals caused failures in compensating for eye movements and increased reliance on post-saccadic sensory feedback to localize visual stimuli [12]. Similar deficits were observed in a visual motion task, where higher autistic quotient (AQ) participants showed reduced sensitivity to motion presented during saccades, a deficit that disappeared when no eye movements were required [13]. Importantly, these impairments appear specific to self-generated movements, suggesting a core difficulty in predicting and integrating internally generated sensory consequences. Further evidence comes from a saccadic adaptation paradigm, where motor learning is triggered by systematic shifts in visual feedback during eye movements. Individuals with high autistic traits and autistics show reduced adaptation in these tasks [14–16], consistent with impaired integration of motor errors. Moreover, when asked to localize targets after finishing saccade execution, high-AQ individuals displayed diminished serial dependencies between motor and perceptual space [15], suggesting that predictive updating failed to generalize across sensorimotor domains, reinforcing the view that they underweight recent experiences when forming new predictions.

Serial dependence has traditionally been characterized as a mechanism that promotes perceptual continuity by smoothing over moment-to-moment variability in sensory input [17–19]. However, emerging evidence suggests that serial dependence extends beyond purely perceptual visual phenomena. Instead, it appears to reflect a more general cognitive and sensorimotor integration process, shaped not only by past stimuli but also by recent responses, decisions, and expectations [20–25]. Recent work shows that serial biases are shaped by task structure and feedback availability, implicating interactions with prediction errors and model updating [26]. Yet it remains unclear how such dependencies operate in dynamic, feedback-rich contexts that approximate real-world action. A key open question is whether trial-history effects in action reflect (i) how accurately forward predictions are calibrated to current contingencies and (ii) how flexibly those predictions are reweighted when environmental statistics change. Dynamic tasks that couple self-generated actions with real-time sensory consequences provide a promising framework to address this. Although emerging studies have begun to examine serial biases in immersive, action-oriented settings [27], few directly manipulate prediction error or measure how feedback shapes internal models across successive trials.

In the present study, we address this gap using a virtual reality (VR) penalty-kick task, designed to simulate a real-world predictive control scenario. Participants kicked a ball that disappeared mid-flight and then estimated its landing point, the position where it would cross the goal line, in a localization task. Crucially, the ball either reappeared in its original trajectory or was laterally shifted to the left or right, creating systematic prediction errors. This manipulation allowed us to derive, on each trial, a prior-informed landing by applying the previous trial’s displacement to the current last-seen ball position and to quantify two complementary aspects of prediction use: (i) prediction deviation, the distance between the response and the prior-informed landing (alignment of the internal prediction with behavior) and (ii) prediction-consistent behavior (β_pred), the extent to which response error increased when responses lay closer to that prior (i.e., how much the prior is relied on, even when it conflicts with current sensory cues). We further computed a directional updating index that captures how much the current correction is carried along the direction of the previous displacement, and we contrasted these model-based measures with signed serial-history slopes (classical stimulus–response history attraction/repulsion). We hypothesized that higher autistic traits would be associated with poorer alignment to prior-informed predictions together with reduced flexibility in reweighting those predictions under increased uncertainty. To contextualize individual-difference effects, we also tested a group of football experts, whose extensive practice was expected to produce stable, well-calibrated sensorimotor mappings that may resist transient, last-trial perturbations [28].

## Results

**Fig. 1:**
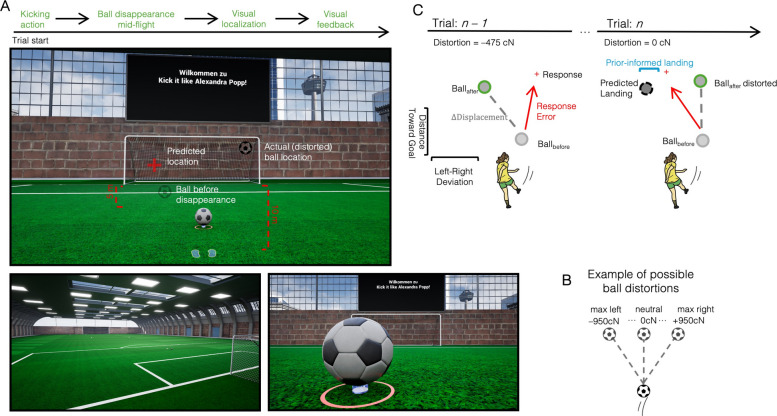
Trial structure and spatial error metrics in the VR penalty-kick task.** A** Trial sequence. Participants performed a kick toward a virtual goal. The ball was visible for a while on its trajectory and disappeared mid-flight (*Ball_before*). Participants provided a manual localization judgment indicating the expected landing position (*Response*) before the ball reappeared and completed its trajectory (*Ball_after*). Crucially, the localization occurred without visual feedback, isolating predictive mechanisms based on internal models and prior sensory cues. (bottom panel, left) A wide-angle third person view of the virtual indoor soccer field is shown. (bottom panel, right). To facilitate shooting behavior in the virtual environment, the ball was positioned slightly above ground level, appearing to float on a small holographic platform. **B** Distortion manipulation. The reappearance of the ball was manipulated by applying lateral forces (distortions) to its trajectory. These could perturb the ball reappearance by lateral forces of − 950, − 475, 0, + 475, or + 950 centiNewtons (cN), producing graded leftward and rightward deviations. Participants experienced the consequences of these distortions only after making their prediction, enabling trial-by-trial updating based on feedback. **C** Goal-plane schematic for two consecutive trials (*n* – 1 to *n*). On trial *n – 1* (left), the ball reappears at position ${\mathrm{B}}_{\mathrm{after}(n-1)}$ that may deviate from its last-seen position ${\mathrm{B}}_{\mathrm{before}(n-1)}$. The participant indicates the expected landing point on the goal plane (red cross). Response error is the goal-plane (X, Y) Euclidean distance between this report and ${\mathrm{B}}_\mathrm{before}$ on that trial. The displacement vector ($\Updelta\mathrm{Distortion}$) captures the trial-specific shift experienced after the response. On trial *n* (right), a prior-informed landing is formed by applying the previous displacement to the current last-seen ball position: $\widehat{\mathrm{L}}_n^\mathrm{prior}={\mathrm{B}}_{\mathrm{before}\left(n\right)}+\Updelta{\mathrm{Distortion}}_{n-1}$ (gray ball). The prediction deviation is the distance between the participant’s response and this prior-informed landing. For completeness, we also compute stimulus history (proximity to the previous post-reappearance position) and response history (proximity to the previous response). These trial-wise components are used in regression models to dissociate passive carryover from flexible prediction updating

**Fig. 2:**
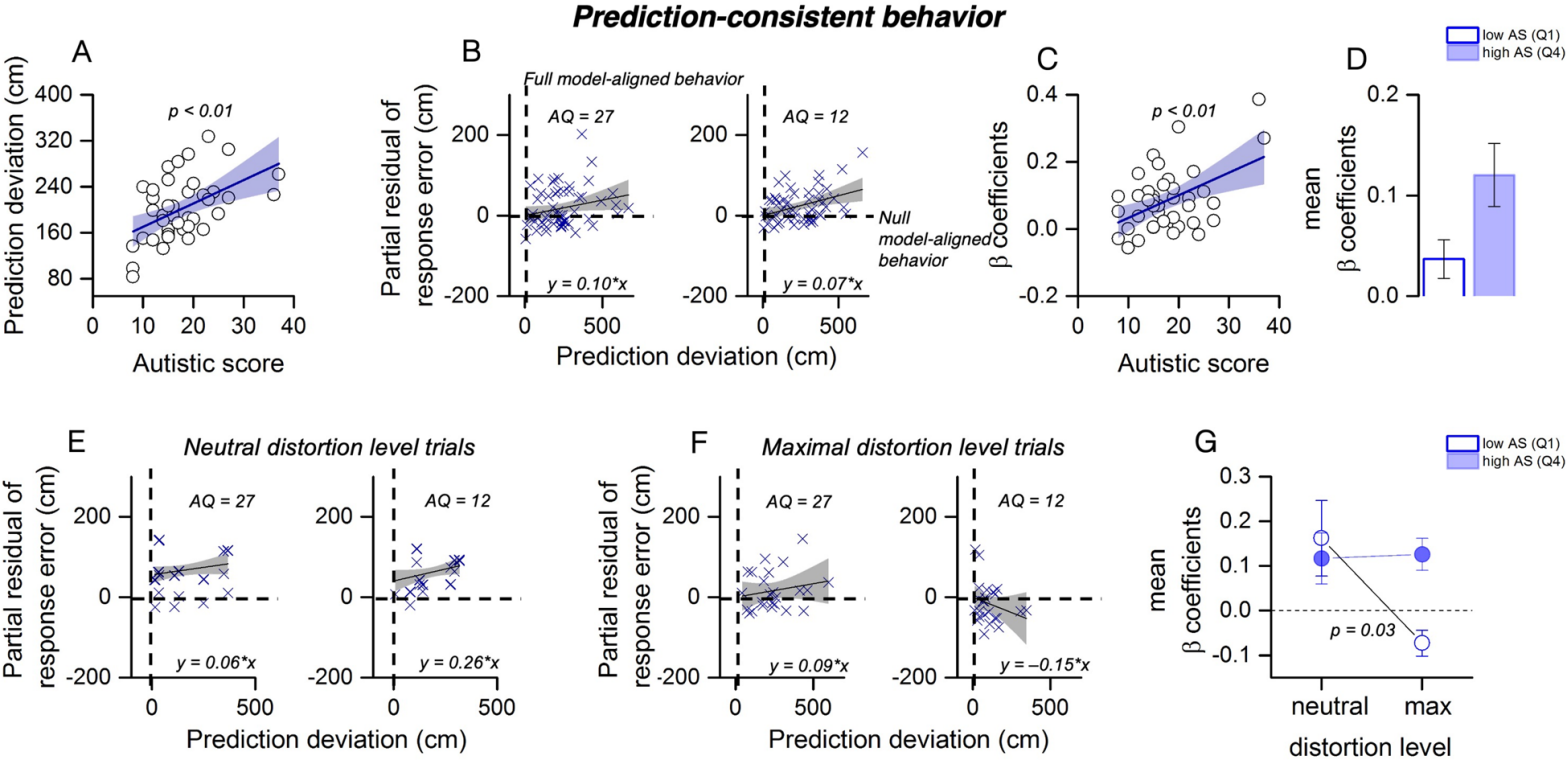
Prediction-consistent behavior***. A*** Prediction deviations (Euclidean distance between response and the prior-informed landing) as a function of autistic quotient (AQ) scores. The thick black line represents the linear regression fit; the shaded area denotes the 95% confidence interval. **B** Trial-level relation between response error and prediction deviation for two example participants with low (AQ = 12) and high (AQ = 27) autistic traits. The steeper positive slope for the high-AQ participant indicates that errors increase as responses move farther from the prior, i.e., being closer to the prior is associated with greater accuracy. This reflects stronger reliance on the prior-informed prediction. The thick black lines show the trial-level regression fits used to extract β coefficients for subsequent analyses. **C** Prediction-consistent slope (β_pred) from each participant’s regression in (B), plotted against AQ score. Linear fit (black line) and 95% confidence interval (shaded) are shown. **D** Group comparison of mean β_pred (low AQ = Q1, high AQ = Q4). Error bars represent ± 1 SEM (standard error of the mean). **E–F** Response error as a function of prediction deviation for two example participants (low AQ = 12, high AQ = 27) across neutral and maximal distortion trials. **G** Mean β_pred split by distortion level (neutral vs. maximum), separately for low- and high-AQ groups. Only low-AQ observers down-weight the prior under maximal distortion. Error bars = ± 1 SEM

Participants performed a VR penalty task, kicking a ball that disappeared mid-flight, and then predicted its landing position with a laser pointer. After each response, the ball reappeared and either continued along its original trajectory or was laterally displaced, creating conditions of low or high environmental uncertainty (Fig. 1A). To quantify how participants integrated recent experience into their predictions, we analyzed several trial-by-trial measures. We first defined a prior-informed landing point by taking the ball’s last-seen position on the current trial and adding the displacement observed in the previous trial. This yielded a predicted landing location based on the assumption that the previous displacement would repeat. We then measured how close participants’ responses were to this point, defining the prediction deviation as the Euclidean distance between the response and the prior-informed landing. Smaller values indicate closer alignment with the prior prediction, whereas larger values indicate departure from it. Second, we asked whether being farther from the prior was associated with larger response errors. Specifically, we regressed response error (distance from the last-seen ball position on the current trial) on prediction deviation. A positive slope means that errors increase as responses move farther from the prior-informed landing: in other words, responses are more accurate when they stay closer to the prior. The slope of this relationship (prediction-consistent behavior, β_pred) indexes reliance on the prior: β_pred > 0 indicates stronger weighting of the prior-based expectation. Figure 2 illustrates these measures as a function of autistic-like traits and environmental uncertainty. The overall prediction deviation was positively correlated with AQ (Fig. 2A, *r* = 0.49, CI = [0.22, 0.70], *p* < 0.01, BF₁₀ = 25.97), indicating that individuals with higher autistic traits were overall less aligned with prior-informed prediction. Importantly, this effect does not appear to reflect a general increase in response variability: the standard deviation of localization errors did not differ between low- and high-AQ participants (*p* = 0.71, BF₁₀ = 0.27), nor did it vary with the continuous of participants’ autistic scores (*r* = 0.15; *p* = 0.36; BF10 = 0.18).

From the trial-by-trial relationship between prediction deviation and current response error, we extracted individual β coefficients indexing reliance on prior expectations (β_Pred, Fig. 2B). These coefficients were positively correlated with AQ scores (*r* = 0.40, CI (confidence interval) = [0.10, 0.63], *p* < 0.01, BF₁₀ = 3.37; Fig. 2C), showing that higher-AQ individuals’ accuracy was more strongly shaped by the prior—even when it conflicted with current ball trajectories. However, direct group comparisons between the first and last quartile of the AQ distribution did not reach significance (t(17) = − 1.71, *p* = 0.10, BF₁₀ = 0.83, Fig. 2D).

Thus, while high-AQ participants were overall less spatially aligned with the prior-informed landing point, their accuracy was more consistently shaped by the prior, reflecting rigid reliance rather than flexible adjustment.

To test whether prior reliance was modulated by environmental uncertainty, we computed β coefficients (β_Pred) separately for trials with no distortion (neutral) and maximal distortion. Example participants’ trial-by-trial data in these conditions are shown in Fig. 2E–F. Under neutral distortion (Fig. 2E), both high- and low-AQ individuals exhibited positive slopes (reliance on the prior). However, under maximal distortion (Fig. 2F), the low-AQ participant markedly downregulated this reliance (slope reversal), whereas the high-AQ participant maintained prediction-consistent behavior. These patterns are confirmed at the group level: a two-way analysis of variance (ANOVA) on prediction dependency slopes (Fig. 2G), with Group (low-AQ vs. high-AQ) and Distortion (neutral vs. maximum distortion) as factors, revealed a significant main effect of Distortion (F(1,62) = 5.00, *p* = 0.03). The main effect of Group was not significant (F(1,62) = 2.57, *p* = 0.11), and the Group × Distortion interaction was not significant (F(1,62) = 3.54, *p* = 0.06). Post hoc *t*-tests indicated that, in the low-AQ group, prediction dependency significantly decreased from neutral to maximum distortion (t(28) = 2.54, *p* = 0.02, BF₁₀ = 2.93). No significant change was observed in the high-AQ group (t(31) = 0.29, *p* = 0.77, BF₁₀ = 0.20). Comparing groups, there was no significant difference under neutral distortion (t(29) = 0.15, *p* = 0.88, BF₁₀ = 0.20), but a significant difference emerged under maximum distortion (t(30) = –4.17, *p* < 0.001, BF₁₀ = 113.96). Together, these results show a pattern consistent with a dissociation: alignment with the prior (prediction deviation) is reduced in high-AQ participants, but reliance on that prior (β_pred) is paradoxically stronger and less flexibly adjusted when environmental uncertainty increases.

**Fig. 3:**
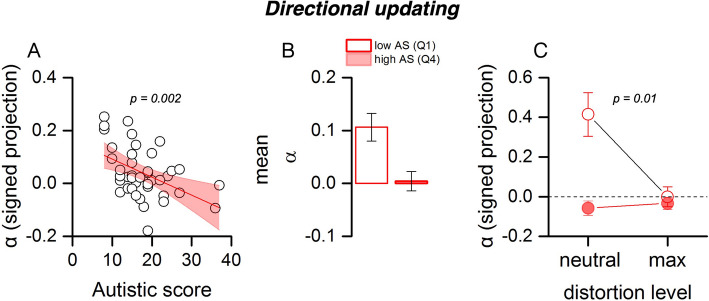
Directional updating. **A** Directional updating index (α; signed projection of the current response onto the previous trial’s displacement) as a function of autistic quotient (AQ) scores. The thick black line represents the linear regression fit; the shaded area denotes the 95% confidence interval. **B** Group comparison of mean α (low AQ = Q1, high AQ = Q4). Error bars represent ± 1 standard error of the mean (SEM). Low-AQ participants show stronger directional updating than high-AQ participants. **C** Mean α values under neutral and maximal distortion, plotted separately for low-AQ and high-AQ groups. Low-AQ participants reduce directional updating under maximal distortion, whereas High-AQ participants remain unchanged. Error bars: ± 1 SEM

Because β_Pred quantifies the magnitude of reliance on the prior-informed prediction but is agnostic to direction, we complemented it with a signed update index, α, that measures directional carryover from the previous displacement (Methods, Eq. [α]). By construction, *α* = 1 indicates full reuse of the preceding displacement along its direction, *α* = 0 indicates no directional carryover, and *α* < 0 indicates repulsion. Across observers (Fig. 3), α decreased with AQ (*r* = − 0.47, 95% CI [− 0.68, − 0.19], *p* < 0.01, BF₁₀ = 14.01), and this association remained when controlling for each participant’s mean displacement magnitude (partial *r* = − 0.57, *p* < 0.001) and precision (partial *r* = − 0.46, *p* = 0.003). Consistent with this trend, low-AQ (Q1) showed larger α than high-AQ (Q4) (t(17) = 2.54, *p* = 0.020, BF₁₀ = 2.84, Fig. 3B). A two-way ANOVA (Fig. 3C) on α with factors group (low vs. high AQ) and distortion (neutral vs. max) revealed main effects of group (F(1,37) = 9.27, *p* = 0.0045) and distortion (F(1,37) = 5.57, *p* = 0.0241), and a group × distortion interaction (F(1,37) = 7.02, *p* = 0.0122). Post hoc tests showed that low-AQ observers down-regulated α from neutral to maximal distortion (t(16) = 2.58, *p* = 0.020, BF₁₀ = 2.99), whereas high-AQ observers did not (t(18) = − 0.38, *p* = 0.705, BF₁₀ = 0.26). Between groups, α differed at neutral (low > high: t(17) = 3.20, *p* = 0.0053, BF₁₀ = 8.73) but not at max (t(17) = 0.43, *p* = 0.670, BF₁₀ = 0.27). The directional updating index showed a pattern consistent with the prediction-reliance measure: participants with higher AQ scores maintained reliance on prior-informed predictions but exhibited reduced directional updating, whereas low-AQ participants flexibly down-regulated both measures under maximal distortion. Thus, the directional updating index complements the prediction-reliance measure by showing that high-AQ participants combine reduced directional carryover (α) with sustained prediction-reliance (β_pred), while low-AQ participants adaptively reduced both.

**Fig. 4:**
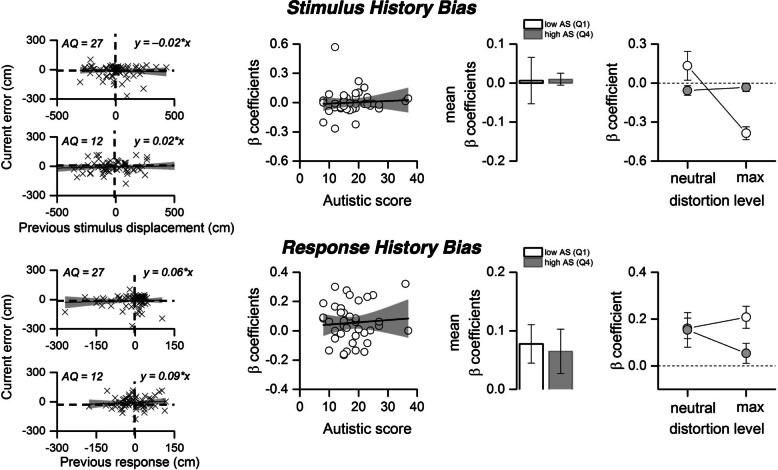
Serial-history biases. **A**, **E** Signed horizontal error plotted against the previous stimulus displacement (stimulus history, **A**) and the previous signed error (response history, **E**) for two example participants (AQ = 12 and AQ = 27). Positive slopes indicate attraction; negative indicate repulsion. Black lines show individual regression fits. **B**, **F** Individual signed slopes (${\beta }_{\mathrm{Stim}}$, ${\beta }_{\mathrm{Resp}}$) as a function of AQ (linear fits with 95% CI). **C**, **G** Group comparisons (low vs. high AQ) for stimulus (**C**) and response (**G**) history biases revealed no differences. **D**, **H** Signed slopes by distortion (neutral vs. maximum), split by AQ group. Error bars: ± 1 SEM

We next examined classical serial dependency measures, stimulus and response dependency, to investigate how participants integrate prior trial information into their current responses. Stimulus dependency quantifies the influence of the previous trial’s stimulus on the current response, while response dependency measures the impact of the participant’s prior response on the current decision. To assess this, we regressed each participant’s signed response errors on the stimulus and response history biases from the preceding trial, yielding individual slope coefficients that quantified how past stimulus and response influences shaped current response errors (Fig. 4). This analysis allowed us to determine whether participants relied more on visual feedback from the environment (stimulus history bias) or their own previous responses (response history bias) when forming their predictions. Example participants with high and low AQ scores are shown in Fig. 4A and E for stimulus and response history, respectively. At the group level, the correlation between stimulus dependency and AQ scores showed no significant relationship (Fig. 4B, *r* = 0.07, *p* = 0.66, BF₁₀ = 0.13), suggesting that the reliance on prior stimulus information did not differ systematically between participants with different levels of autistic traits. Similarly, response dependency was not significantly correlated with AQ scores (Fig. 4F, *r* = 0.08, *p* = 0.63, BF₁₀ = 0.14), indicating that the influence of prior responses on current actions was not strongly related to AQ. For both measures, we also compared the first and last quartile of the AQ distribution. For stimulus dependency, the comparison showed no significant difference (Fig. 4C, t(17) = 0.19, *p* = 0.84, BF₁₀ = 0.01). Similarly, response dependency also showed no significant difference between the first and last quartiles of AQ (Fig. 4G, t(17) = − 0.05, *p* = 0.96, BF₁₀ < 0.01). One-sample *t*-tests against zero further confirmed that neither group showed significant attraction or repulsion effects (response-history: low-AQ *p* = 0.11, high-AQ *p* = 0.21; stimulus-history: low-AQ *p* = 0.62, high-AQ *p* = 0.94). A two-way ANOVA on the signed stimulus-history slopes (Fig. 4D, H) with factors Group (low vs. high AQ) and distortion (neutral vs. maximum) revealed no main effects (group: F(1,28) = 0.32, *p* = 0.5781; distortion: F(1,28) = 0.66, *p* = 0.4236) and no interaction (F(1,28) = 1.78, *p* = 0.1947). These analyses indicate that neither AQ group nor distortion level reliably modulated classical signed serial-dependence.

**Fig. 5:**
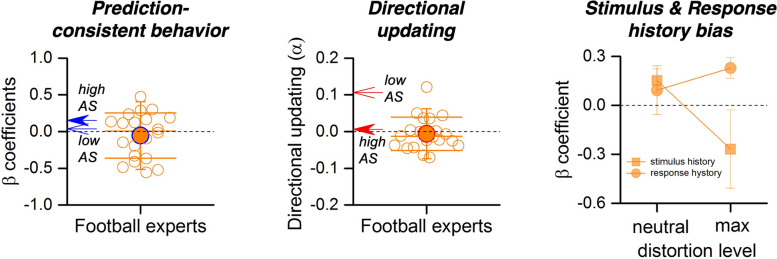
Prediction reliance, directional updating, and signed history biases in football experts. **A** Prediction-reliance slopes (β_pred) for individual experts (orange circles). The large, filled circle shows the group mean. For comparison, mean values for low–AQ and high–AQ groups are indicated by blue arrows. **B** Directional updating index (α) for experts. Orange circles show individual participants; the filled circle indicates the group mean. Red arrows mark mean values for low–AQ and high–AQ groups. **C** Signed history biases in experts, plotted separately for neutral and maximal distortion. Squares show stimulus-history slopes; circles show response-history slopes. Error bars: ± 1 SEM

To characterize how football expertise shapes trial-history use and prediction, we analyzed a dedicated subsample of trained players (≥ 4 years practice), focusing on prediction-consistent behavior (β_pred), directional updating (α), and signed stimulus- and response-history slopes across distortion levels (Fig. 5). To ensure that any differences reflect updating rather than generic performance, we first verified that baseline accuracy and precision were comparable across groups. Mean response error and response-error variability did not differ between low-AQ, high-AQ, and experts (accuracy: ANOVA *p* = 0.470; Kruskal–Wallis *p* = 0.675; precision: ANOVA *p* = 0.657; Kruskal–Wallis *p* = 0.657). In experts, β_Pred (Fig. 5A) did not differ from zero and showed no distortion dependence (β_Pred vs. 0: *p* = 0.95, BF₁₀ = 0.24; neutral vs. max: *p* = 0.30, BF₁₀ = 0.39) and did not differ by group after adjustment (low–high *p* = 0.560; low–expert *p* = 0.331; high–expert *p* = 0.090), indicating that the group effect is specific to very recent directional use of the last distortion rather than to generic weighting of predictions. Directional flexibility index α (Fig. 5B) was significantly reduced in experts relative to low-AQ observers (*p* = 0.003, *d* = 1.52) and statistically indistinguishable from high-AQ observers (*p* = 0.67, *d* = 0.17). These results held after covarying for accuracy, precision, and the average displacement magnitude (analysis of covariance (ANCOVA): low > high, *p* = 0.005; low > expert, *p* = 0.003; high = expert, *p* = 0.965).

By contrast, signed serial dependence (Fig. 5C) revealed no stimulus-history carryover (β_Stim: *p* = 0.90, BF₁₀ = 0.23) but a positive response-history slope (β_Resp: *p* = 0.017, BF₁₀ = 3.20), consistent with response inertia; across distortion, β_Stim decreased from neutral to maximal (*p* = 0.047, BF₁₀ = 1.83) whereas β_Resp was unchanged (*p* = 0.53, BF₁₀ = 0.39). Together, these results indicate that experts share the low-α (reduced directional updating) profile observed in High-AQ participants despite matched accuracy and precision, while β_Pred remains near zero; additionally, experts exhibit stable response inertia and a modest distortion-dependent change in stimulus-based carryover.

## Discussion

Our study employed a dynamic VR penalty‐kick task in which participants kicked a ball that disappeared mid-flight and then localized its expected landing position before the ball reappeared. This design simulated a real‐world sensorimotor scenario requiring continuous integration of internal predictions and sensory feedback. By varying the ball’s trajectory and computing, on each trial, a prior-informed landing (the current last-seen position plus the previous trial’s displacement), we examined how participants integrated prior feedback into their ongoing responses, and how this process varied as a function of autistic-like traits and expertise. Critically, we found that the distance between participants’ responses and this prior-informed landing (the prediction deviation) was significantly larger in individuals with higher AQ scores. This indicates that their responses were less aligned with predictions based on recent sensory history. Importantly, this difference did not reflect a general increase in variability or motor noise, as overall response dispersion was comparable across groups. Instead, it suggests a reduced tendency, or capacity, to incorporate recent outcome information into forward models, even in stable environments. To quantify the extent to which participants relied on these predictions, we related Response Error to Prediction Deviation and extracted a prediction-reliance slope (β_pred). Larger positive β_pred values mean that errors increase as responses move farther from the prior-informed landing—that is, accuracy is higher when participants stay close to the prior. This reflects stronger reliance on the prior, even when it no longer matches the current ball trajectory. We found that this prediction-consistent behavior was more pronounced in individuals with higher AQ scores. In other words, high-AQ participants tended to respond in line with what they had implicitly learned from previous outcomes, even when those predictions no longer matched the actual ball trajectory. By contrast, low-AQ individuals appeared more flexible, adjusting their reliance on these internal expectations when the environment changed.

This interpretation was supported by our distortion-level analyses: low-AQ participants reduced prediction-reliance (β_pred) under maximal distortion, whereas high-AQ participants showed no such adjustment. This group-specific modulation implies reduced context-sensitive reweighting of sensory evidence versus priors at higher AQ, consistent with predictive-coding accounts proposing attenuated precision modulation in autism [10, 26]. Rather than indicating a failure to form predictions per se, our findings suggest reduced flexibility in how internal predictions are weighted and deployed when uncertainty changes.

Mechanistically, our results point to a dissociation in high-AQ observers between model calibration and model weighting. First, larger Prediction Deviation indicates that the prior-informed landing is less well aligned with the current trajectory (poorer calibration), potentially due to imprecise efference-copy/state estimates or mis-scaling of the previous displacement across contexts [11, 13]. Second, the prediction-consistency index (β_pred) remains elevated and fails to down-shift under maximal distortion, indicating that these suboptimal predictions continue to be granted high weight even when the environment becomes volatile. In predictive-coding terms, this pattern is consistent with attenuated precision modulation of recent priors [10, 29]: prediction errors do not sufficiently reduce the influence of a mismatched prior, yielding poorer alignment coupled with rigid reliance.

Complementing the prediction-reliance measure, we quantified a directional updating index that captures how much the current correction is carried along the previous trial’s displacement. This index was smaller at higher AQ and, critically, decreased under maximal distortion only in low-AQ observers, indicating context-sensitive down-weighting of the last displacement when it becomes unreliable. High-AQ observers, by contrast, did not modulate directional updating with distortion. Taken together with the prediction-reliance result above, this pattern suggests a dissociation in high-AQ participants: they maintain reliance on recent predictions even when these conflict with current input, while updating less in the appropriate direction from one trial to the next. Because these effects persist after controlling for accuracy, precision, and displacement magnitude, they are unlikely to reflect generic noisiness and instead point to reduced flexibility in re-allocating weight between recent priors and sensory evidence when environmental statistics change. By contrast, experts showed low directional updating together with near-zero prediction-consistency (β_pred), suggesting a well-calibrated mapping that is deliberately down-weighted against last-trial perturbations, stability rather than sticky mis-calibration, despite matched accuracy and precision across groups.

While classical serial dependence effects, i.e., biases toward previous stimuli or responses, have been widely documented in perceptual tasks [17], we found little evidence for these effects in our motor prediction task. Across the main sample, neither stimulus dependency nor response dependency was significantly modulated by autistic traits, distortion level, or their interaction. This suggests that the serial structure we observe is not driven by traditional carryover, but instead reflects reliance on internally generated predictions updated by external feedback, in line with accounts in which action-related serial biases arise from error monitoring and expectation updating alongside any passive carryover [27].

Our findings also highlight the role of domain expertise in shaping internal predictive processes. Experts exhibited reduced directional updating from one trial to the next, near-zero prediction-reliance (i.e., no generic pull toward the prior-informed prediction), and a signed history profile marked by response inertia without stimulus-history attraction, with the latter decreasing under maximal distortion. Accuracy and precision were matched across groups, indicating that these differences reflect information weighting rather than generic noisiness. This pattern is consistent with robust, overlearned sensorimotor mappings that resist transient, last-trial perturbations and rely on predictive structures accrued over longer times. Rather than flexibly adjusting predictions from trial to trial, experts appear to maintain well-calibrated mappings that are not substantially modulated by brief feedback perturbations [28]. By contrast, high-AQ participants combine reduced directional updating with sustained prediction-reliance and little context-dependent reweighting, a profile more consistent with rigid deployment of recent predictions than with strategic robustness. Thus, superficially similar reductions in directional updating arise from different mechanisms: overlearned stabilization in experts versus inflexible prediction use at higher AQ.

In addition to differences in predictive weighting, the ability to evaluate the reliability of one’s own predictions may further shape adaptive behavior. Confidence has been proposed to act as an internal estimate of certainty that determines how strongly prior information is weighted against new evidence [29]. Because higher confidence amplifies serial-dependence biases [30], future work could test whether group differences in predictive weighting reflect altered confidence calibration.

Finally, although our task did not directly isolate motor-based prediction errors, the position of the ball before disappearance was determined by the participant’s own kick. Thus, anticipating the ball’s trajectory inherently involved estimating the outcome of a self-generated action. In real-world scenarios, such estimations are thought to rely on efference copy mechanisms, internal signals that represent planned motor output and enable forward prediction. While our model-derived predictions were based on previous trial distortions rather than motor output per se, reduced alignment and updating in individuals with higher AQ may reflect broader inefficiencies in forming and adjusting predictions about the sensory consequences of action. This interpretation is consistent with prior evidence linking autistic traits to imprecise efference copy signals during saccadic and motor tasks [11, 13].

## Conclusions

Taken together, our results support a view of serial dependence as a multifaceted process, one that includes but extends beyond perceptual smoothing. In dynamic, goal-directed contexts, serial dependencies reflect not just passive carryover, but the flexible, or rigid, deployment of internally generated predictions to guide behavior. By quantifying how closely participants’ responses aligned with predictions informed by prior outcomes, we demonstrate that prediction-consistent behavior varies systematically with autistic traits and expertise. These findings contribute a novel perspective to predictive coding accounts: highlighting not only how predictions are formed, but how they are selectively used, and sometimes misused, during action. This distinction may be critical for understanding real-world impairments in sensorimotor integration in autism.

## Methods

### Participants

Seventy-one healthy participants (age, 18–35, 45 females) voluntarily participated in the study and provided written consent after receiving detailed information. All participants had normal or corrected-to-normal vision. Neurological and psychiatric disorders, brain injuries, movement disorders, and the use of central nervous system medications were exclusion criteria. One participant was excluded from the data analysis due to a brain tumor in the past, one because of an impaired working memory, and eight others because of missing data due to technical problems during the experiment. The final sample consisted of 42 participants, plus 19 soccer experts.

### Apparatus

The virtual reality (VR) environment was developed using Unreal Engine (version 4.27). Participants wore an HTC Vive Pro Eye Head-Mounted Display (HMD; resolution: 1440 × 1600 pixels per eye, 90 Hz refresh rate, about 110° horizontal field of view) connected via SteamVR 2.0 to an Alienware Aurora R8 desktop (Intel Core i7-8700, 3.2 GHz, 16 GB RAM, NVIDIA RTX 2080 GPU). Hand and finger movements were tracked with Valve Index controllers (represented visually as floating gloves), while Vive trackers attached to participants’ feet provided accurate tracking of kicking movements (represented visually as soccer shoes).

Eye movements were recorded at 120 Hz using the built-in HTC Vive Pro Eye tracking system and analyzed with the SRanipal Application Programming Interface (API) [31].

### Procedure

Each participant was equipped with two hand trackers, two foot-trackers, and a tracked HMD. They were instructed by the experimenter to look around the virtual soccer stadium and familiarize themselves with the set-up. To reduce the number of missed shots, the size of the virtual goal was substantially increased compared to standard football dimensions. Specifically, the goal measured 20 m in width and 5.7 m in height, considerably larger than a regulation goal (7.32 m wide and 2.44 m high). This adjustment ensured that participants, regardless of their athletic background, could engage effectively with the task and that performance variability would more likely reflect differences in prediction and updating rather than basic motor execution or accuracy limitations. In contrast, the virtual ball was modeled at standard scale, with a diameter of 22 cm, corresponding to the size of a regular soccer ball.

The instructions were displayed on a screen above one of the goals and supplemented with verbal explanations from the experimenter (see Fig. 1). To become familiar with virtual soccer playing, some practice free-shoot trials were conducted until the participants felt ready to start with the actual task. Subsequently, 20 trials were conducted, during which the participants had to both shoot and localize a ball in the goal. Afterwards, the experiment continued with 125 trials where participants had to shoot and localize the ball, with visual distortion of the ball applied.

At the beginning of each trial, participants were instructed to stand on footprints (included to standardize participants’ starting position) placed at 10 m from the goal and then asked to look at a circle positioned one meter ahead of them on the ground. This fixation triggered the appearance of a soccer ball; participants were asked to kick the ball into the goal and observe the trajectory. During its flight, the ball passed through an invisible collision volume positioned 5 m in front of the goal. Upon entering this zone, it paused after a variable delay between 0 and 200 ms. Participants then had to fixate on the ball to proceed, ensuring they had seen its last location before it disappeared. Participants were then instructed to use a laser to mark the point where they believed the ball would land in the goal. To confirm their localization response, participants were instructed to press the trigger button on the controller *not* used for pointing with the laser (Fig. 1A, upper panel). This setup was chosen to minimize motor interference between the act of confirming the response and the pointing gesture itself. Pressing the trigger on the same hand used for aiming could have introduced small, unintended movements, potentially displacing the laser and compromising responses. By requiring the use of the opposite hand for confirmation, we ensured that the pointing position remained stable during response registration. After marking the point, the ball reappeared and continued its trajectory, either following the natural continuation (distortion neutral, or zero) or by being deviated more to the left or to the right (distortion max). Five different levels of this manipulation were applied: one larger and one smaller distortion to the left, one with no distortion and one larger and one smaller distortion to the right (Fig. 1B). A custom randomization algorithm ensured that distortions were never repeated more than twice consecutively.

To facilitate shooting behavior in the virtual environment, the ball was positioned slightly above ground level, appearing to float on a small holographic platform (Fig. 1A, bottom right panel). This design choice addressed a key limitation of virtual environments: unlike in the real world, where the ground, ball, and footwear deform slightly upon contact, all three are modeled as rigid bodies in VR. As a result, realistic interactions, such as sliding the foot under the ball to lift it into the air, are difficult to reproduce. In reality, subtle deformations allow the foot to wedge slightly beneath the ball when executing a chip or lofted shot. Without such flexibility, the foot in VR often collides with the ball’s lower edge, impeding natural kicking dynamics. Elevating the ball marginally above the ground resolved this issue by making it easier for participants to make clean contact from below.

### Autistic quotient (AQ) and expertise

All participants completed the self-administered Autistic Quotient questionnaire (validated German or English versions; [32, 33]). The AQ consists of 50 items grouped into five subscales: attention switching, attention to detail, imagination, communication, and social skills. For each item, participants indicated agreement or disagreement with presented statements, resulting in a binary scoring (0 or 1). Total scores ranged from 0 to 50, with higher scores indicating stronger autistic traits. In our sample, AQ scores had a median of 16 (lower quartile = 12.75, upper quartile = 22). The distribution of AQ scores did not deviate from normality (Jarque–Bera test: JB = 1.31, *p* = 0.31). All participants scored below 32, the threshold above which clinical assessment is recommended, except two participants who scored above 36 but reported no diagnosis at the time of testing. To examine whether autistic traits were related to task performance, correlation analyses were conducted between individual AQ scores and the measured variables. Additionally, participants were divided into groups based on AQ scores, comparing performance between participants in the lower quartile (Q1) and upper quartile (Q4).

Nineteen participants (age *M* = 21.65, SD = 2.80) were classified as soccer experts, based on their competitive play history and the extent of their training (4–12 years of experience). To evaluate whether soccer expertise influenced performance, analyses were repeated with “expertise” included as a between-subjects factor in ANOVAs, and post hoc *t*-tests were conducted where appropriate.

### Data analyses

To quantify serial dependency and sensory–motor predictions in our VR penalty–kick task, we computed several trial-by-trial error metrics (Fig. 1C).

*Response error* (goal-plane distance to last-seen position). Participants indicated the point on the goal plane where they expected the ball to land. For each trial *n*, response error was computed as the Euclidean distance in the goal plane between the reported location ${L}_{n}=({L}_{x(n)},{L}_{y(n)}$) and the ball’s last-seen position before disappearance ${B}_{\mathrm{before}(n)}=({B}_{x,\mathrm{before}(n)},{B}_{y,\mathrm{before}(n)}$)

$${\mathrm{ResponseError}}_{n}=\left|{L}_{n}-{B}_{\mathrm{before}(n)}\right|= \sqrt{{\left({L}_{x,n}-{B}_{x,\mathrm{before}(n)}\right)}^{2}+{\left({L}_{y,n}-{B}_{y,\mathrm{before}(n)}\right)}^{2}}$$

The following key metrics were computed as predictors:

Prediction deviation (distance to prior-informed landing).

To test reliance on short-term internal predictions, we first compute the previous-trial displacement (the reappearance shift) as.

1 $$\Updelta{\mathrm{Distortion}}_{n-1}\;=\;B_{\mathrm{after}\left(n-1\right)}\;-\;B_{\mathrm{before}\left(n-1\right)}$$

where ${B}_{\mathrm{before}(n-1)}$ is the ball’s last-seen position before disappearance on trial n–1 and ${B}_{\mathrm{after}(n-1)}$ is its post-reappearance position in the goal plane.

For the current trial *n*, we form a prior-informed landing by applying that displacement to the current last–seen position:

$$\widehat{\mathrm L}_{\mathrm n}^{\mathrm{prior}}\;=\;{\mathrm B}_{\mathrm{before}\left(n\right)}\;+\;\triangle{\mathrm{Distortion}}_{\mathrm n-1}$$

The prediction deviation is then the Euclidean distance between the participant’s reported landing ${L}_{n}$ and this prior-informed landing:

$${\mathrm{PredictionDeviation}}_{n}= \left|{L}_{n}-{\widehat{\mathrm{L}}}_{\mathrm{n}}^{\mathrm{prior}}\right|= \sqrt{{\left({L}_{x,n}-{\widehat{\mathrm{L}}}_{\mathrm{n}}^{\mathrm{prior}}\right)}^{2}+{\left({L}_{y,n}-{\widehat{\mathrm{L}}}_{\mathrm{n}}^{\mathrm{prior}}\right)}^{2}}$$

Intuitively, smaller prediction deviation reflects greater alignment with the prior-informed landing (the previous displacement applied to the current trajectory), whereas larger values reflect greater departure from that prior.

By itself, this is a geometric proximity measure; we therefore assess reliance on the prior via the regression coefficient β_Pred (see Regression Modelling) and capture direction with the signed projection α.

### Directional projection ($\alpha$)

To capture direction and amount of carry-over from the previous trial’s displacement, we projected the current response (relative to the current last-seen position) onto the previous displacement in the goal plane (x, y):

$$\alpha_n\;=\;\frac{{(\mathrm{L}}_\mathrm{n}-{\mathrm{B}}_{\mathrm{before}\left(n\right)})\times{(\mathrm{B}}_{\mathrm{after}(n-1)}-{\mathrm{B}}_{\mathrm{before}\left(n-1\right)})}{\vert{{(\mathrm{B}}_{\mathrm{after}(n-1)}-{\mathrm{B}}_{\mathrm{before}\left(n-1\right)}\vert}^2}for\vert B_{\mathrm{after}(n-1)}\;-\;B_{\mathrm{before}(n-1)}\vert\;>\;0.$$

Intuitively, α quantifies the fraction of the previous displacement reproduced on the current trial along its direction: *α* = 0 indicates no carry-over (e.g., aiming at the undistorted location); *α* = 1 indicates full carry-over (aiming at the displacement-shifted location); *α* > 1 indicates over-compensation; and *α* < 0 indicates repulsion (bias in the opposite direction).

#### Proximity covariates (stimulus/response proximity)

Stimulus proximity quantifies the proximity of the current response to the previous trial’s stimulus (post-reappearance) position. It is computed as the Euclidean distance between the current localization ${L}_{(n)}$ and the ball’s post-reappearance position in the previous trial ${B}_{\mathrm{after}(n-1)}$:

$$\text{Stimulus Proximity}=\sqrt{{\left({L}_{\left(n\right),x}-{B}_{\mathrm{After}\left(n-1\right),x}\right)}^{2}+{\left({L}_{\left(n\right),y}-{B}_{\mathrm{After}\left(n-1\right),y}\right)}^{2}}$$

Response proximity quantifies the proximity of the current response to the participant’s previous response. It is computed as the Euclidean distance between the current and previous localization:

$$\text{Response Proximity}=\sqrt{{\left({L}_{(n),x}-{L}_{(n-1),x}\right)}^{2}+{\left({L}_{(n),y}-{L}_{(n-1),y}\right)}^{2}}$$

These quantities are non-negative proximity measures and are used only as covariates in the multivariate prediction model to account for trial-wise closeness to the previous stimulus/response. Directional serial-dependence effects (attraction vs. repulsion) are assessed separately via the signed horizontal analyses described below.

#### Directional serial dependence (signed horizontal analyses)

To assess direction along the distortion axis (*X*), we computed:

i. The signed previous stimulus displacement
  $$\Updelta{\mathrm x}_{\mathrm n-1}\;=\;{\mathrm{Bx}}_{\mathrm{after}(\mathrm n-1)}\;-\;{\mathrm{Bx}}_{\mathrm{before}\left(\mathrm n-1\right)}$$
ii. The signed previous and current error
  $${\mathrm{Errx}}_{\mathrm{n}-1}= {\mathrm{Lx}}_{\left(\mathrm{n}-1\right)}- {\mathrm{Bx}}_{\mathrm{before}\left(\mathrm{n}-1\right)}$$
  $${\mathrm{Errx}}_{\mathrm{n}}= {\mathrm{Lx}}_{\left(\mathrm{n}\right)}- {\mathrm{Bx}}_{\mathrm{before}\left(\mathrm{n}\right)}$$

We then fit simple regressions.

$${\mathrm{Errx}}_{\mathrm n}\;=\;{\mathrm\beta}_0\;+\;{\mathrm\beta}_{\mathrm{Stim}}\;\Updelta{\mathrm x}_{\mathrm n-1}\;+\;\mathrm\varepsilon$$

$${\text{Err x}}_{\mathrm{n}}={\upbeta }_{0}+{\upbeta }_{\mathrm{Resp}}\cdot {\text{Err x}}_{\mathrm{n}-1}+\upvarepsilon$$

With this convention, positive β_Stim or β_Resp indicates attraction (the current signed error shifts in the same horizontal direction as the previous stimulus/response), and negative slopes indicate repulsion. We refer to these as the signed stimulus-history slope (β_Stim) and the signed response-history slope (β_Resp). These signed slopes are used for AQ correlations, group comparisons, and distortion-level analyses. The unsigned proximity covariates are used only for adjustment in the multivariate prediction model. These signed slopes are used for AQ correlations and group comparisons.

#### Regression modelling

To quantify reliance on recent information, we regressed response error on three predictors: *StimulusHistoryBias*, *ResponseHistoryBias*, and *PredictionDeviation.*

1. Prediction Model
  $${\text{Response Error}}_{n}={\beta }_{0}+{\beta }_{\mathrm{StimHist}}\cdot \left(\mathrm{StimulusHistoryBias}\right)+{\beta }_{\mathrm{RespHist}}\cdot \left(\mathrm{ResponseHistoryBias}\right)+{\beta }_{\mathrm{Prediction}}\cdot {\mathrm{PredictionDeviation}}_{n}+\varepsilon$$

Here, ${\beta }_{0}$ is the intercept and $\varepsilon$ the error term. A ${\beta }_{\mathrm{Prediction}}>0$ indicates that when participants deviate from the prior-informed landing, their errors increase, i.e., responses are more accurate when closer to the prior. This implies reliance on the prior. A ${\beta }_{\mathrm{Prediction}}<0$ means that deviating from the prior reduces error, consistent with down-weighting the prior under instability. ${\beta }_{\mathrm{StimHist}}$ and ${\beta }_{\mathrm{RespHist}}$ capture magnitude effects of stimulus and response history biases, respectively. We also fit the model separately for neutral vs. maximum distortion to test whether reliance on prior-based predictions varies with environmental uncertainty.

For visualization only, we plot partial residuals to show the unique association between each predictor and the outcome after adjusting for the others. For a regression

$${r}_{X,n}^{(\mathrm{partial})}={\widehat{\varepsilon }}_{n}+{\widehat{\beta }}_{x}{X}_{n} for X \in \{\mathrm{StimulusHistoryBias},\text{ResponseHistoryBias },\text{ PredictionDeviation}\}$$

Partial residuals can take negative values due to residual centering, even though the underlying distances (predictors and response error) are non-negative.

To verify that our sequential dependency measures were not artifacts introduced by the bias‐correction procedure, for each participant we randomly shuffled the order of trials and refit our model to these shuffled data (as in [25]). In our analysis, the mean shuffled regression slope was − 0.013 and the mean *p*-value was 0.498. These results confirm that the sequential effects observed in our original (non-shuffled) data are genuine and not an artifact.

For each subject, these three regression models were each fit to raw data excluding outliers of 2.5 SD from the mean response error and trials in which the ball either rolled on the floor or ended up outside the goal. After removing the confounding bias in the raw data, we excluded the first trial of every block, since it does not have any preceding trial to induce sequential effects and the subsequent trials on which subjects could have been affected by those preceding trials with outlying responses. In total, 8% of the trials were excluded due to this outlier correction.

A preliminary analysis on distortion levels indicated no significant difference between the left and right distortions in any of the variables tested (all *p* > 0.10); hence, these two were collapsed into a single “max” condition. The “neutral” condition corresponded to 0 centiNewtons (cN) distortion.

Repeated-measures ANOVAs to test the effects of factors such as distortion conditions and AQ groups (high vs. low AQ, based on quartiles) on response errors and on extracted regression slopes were run. Significant interactions were followed by post hoc comparisons using paired *t*-tests, adjusted for multiple comparisons where necessary. For each participant, ${\mathrm\alpha}_\mathrm{n}$ was averaged across eligible trial pairs and tested (i) correlation with AQ, (ii) group differences (low-AQ vs. high-AQ; experts), and (iii) an ANCOVA controlling for accuracy (mean response error), precision (standard deviations of response error), and mean displacement magnitude.

To quantify the strength of evidence supporting or rejecting our hypotheses, we performed Bayesian analyses. Bayes Factors, expressed as ratios comparing likelihoods of alternative versus null hypotheses, were computed for key comparisons. Bayes factors (BF₁₀) greater than 3 are considered moderate evidence for the alternative hypothesis; values below 1/3 support the null [34, 35].

## Data Availability

All data generated or analysed during this study are included in this published article, its supplementary information files and publicly available repositories [36].

## Abbreviations

AQ: Autistic quotient
ASD: Autism spectrum disorder
BF₁₀: Bayes factor
cN: CentiNewton
CI: Confidence interval
HMD: Head–Mounted display
OSF: Open Science Framework
SD: Standard deviation
SEM: Standard error of the mean
VR: Virtual reality
API: Application programming interface
ANOVA: Analysis of variance
ANCOVA: Analysis of covariance
